# Blockade of Gi Signaling Enhances the Anabolic Effect of Parathyroid Hormone in Female Mice

**DOI:** 10.1007/s00223-025-01409-2

**Published:** 2025-07-16

**Authors:** Liping Wang, Lalita Wattanachanya, Vikrant Piprode, Robert A. Nissenson

**Affiliations:** 1 Endocrine Research Unit, VA Medical Center, San Francisco, CA USA; 2 Department of Medicine, University of California, San Francisco, CA USA; 3 Division of Endocrinology and Metabolism, Department of Medicine, Faculty of Medicine, and Excellence Center for Diabetes, Hormone, and Metabolism, Chulalongkorn University, King Chulalongkorn Memorial Hospital, Bangkok, Thailand

**Keywords:** PTH, Anabolic action, Gi signaling, GPCR, Bone, G protein-coupled receptor, Gi signaling, Anabolic, Parathyroid hormone

## Abstract

Intermittent administration of parathyroid hormone (PTH) produces potent anabolic effects on bone. These anabolic effects appear to be mediated by activation of the Gs-cyclic AMP system initiated by the PTH1 receptor (PTHR1) in osteoblast lineage cells. Gs-mediated cyclic AMP production can be inhibited by activation of Gi signaling, and we have previously demonstrated that Gi signaling in osteoblasts suppresses bone formation and thereby plays a prominent role in the bone loss associated with aging in female mice. It is not clear whether this increased Gi signaling with aging dampens the anabolic response to intermittent PTH (iPTH) administration. To address this possibility, we determined the effect of inducible blockade of Gi signaling in osteoblasts on the anabolic response to PTH in 4-month-old female mice. Blockade of Gi signaling was achieved by tetracycline-regulated expression of pertussis toxin (PTX) driven by a Col1(2.3) promoter. We found that the expression of PTX potentiated the anabolic effect of iPTH on the trabecular bone, both at the distal femur and L4 lumbar vertebra. In addition, the ability of PTH to promote cortical bone thickness and strength was evident only in mice expressing PTX. These novel results demonstrate that endogenous Gi signaling in osteoblasts limits the anabolic action of iPTH on the skeleton of adult female mice. Strategies that suppress osteoblast Gi signaling could provide an effective adjunct to iPTH therapy in the treatment of postmenopausal osteoporosis.

**Supplementary Information:**

The online version contains supplementary material available at 10.1007/s00223-025-01409-2.

## Introduction

Parathyroid hormone (PTH) is one of a few anabolic drugs approved by the FDA to restore bone structural integrity and to reduce fracture risk in people with osteoporosis. The ability of intermittent PTH (iPTH) to increase bone formation over resorption accounts for its anabolism. However, there are some limitations in the clinical utility of PTH as an anabolic agent. There is a reduction in the anabolic response to PTH after an initial anabolic phase [1] and PTH has a limited anabolic effect on non-vertebral bones and cortical bone [2, 3]. Thus, the ability to enhance the therapeutic efficacy of iPTH treatment would have considerable clinical impact.

PTH/PTHrP receptor 1 (PTH1R) is highly expressed in bone. It couples to several G protein subclasses, including G_s_, G _q/11_, and G _12/13_. PTH produces bone anabolic effects mainly by binding to G_s_ protein, activating G_s_ signaling pathway that results in cyclic AMP (cAMP) synthesis, protein kinase A (PKA) activation, and bone formation [4, 5]. The anabolic response of G_s_ requires the Wnt signaling pathway, which plays a crucial role in osteoblast differentiation and function [6]. In pre-osteoblasts, canonical WNT signaling stimulates replication and promotes osteoblast differentiation [7], while in mature osteoblasts, canonical WNT signaling regulates bone remodeling by secreting osteoprotegerin (OPG) to block osteoclastogenesis [8]. When Wnt signaling is suppressed with DKK1, a Wnt receptor antagonist, PTH-mediated peri-trabecular bone stromal cell recruitment, and new bone formation are reduced [9]. Blockade of the Wnt signaling pathway in osteoblasts and osteocytes prevents the iPTH-induced bone gain in mice [10]. However, to what extent PTH requires Wnt signaling in osteoblast lineage cells to produce a profound anabolic effect is still not clear.

In contrast to G_s_ protein, constitutive activation of G_i_ signaling in mouse osteoblasts has been demonstrated to cause marked osteopenia [11]. A specific blockade of G_i_-coupled GPCR signaling in osteoblasts by expressing the pertussis toxin (PTX) gene not only increases both cortical and cancellous bone in mice [12] but also prevents age-related bone loss in female mice [13]. Some studies have also shown that G_i_-GPCRs and their signaling pathways may be linked with osteoporosis in humans as well. For instance, the level of plasma sphingosine 1-phosphate (S1P), a ligand for its G_i_ protein-coupled receptor (S1P1), is inversely correlated with bone mineral density, and positively correlated with bone resorption markers [14, 15]. These data indicate that endogenous G_i_ signaling restrains bone formation in adult mouse bone. Furthermore, PTH1R also couples to G_i_ protein, and thus, activation of PTH1R-Gi may compromise PTH1R-G_s_-mediated increase in adenylyl cyclase activity [16]. We therefore hypothesized that blockade of G_i_ signaling in osteoblasts would enhance the bone anabolic effect of the PTH.

To study the effects of activation of G_i_ signaling in osteoblasts, we have established a mouse model, in which the catalytic subunit of PTX gene is expressed under the control of the 2.3 kb Collagen type I-tTA (Col1(2.3)-tTA) promoter (Col1(2.3)^+^/PTX^+^ mouse) [12, 13]. To test our hypothesis, we treated the adult Col1(2.3)^+^/PTX^+^ mice with intermittent PTH and then characterized bone structure and mechanical properties. Furthermore, To understand the mechanisms of endogenous G_i_ signaling blunting the anabolic effect of PTH, we performed a gene microarray to assess the gene profile of the Col1(2.3)^+^/PTX^+^ mouse osteoblasts and carried out RNA-Seq to assess the transcriptome profile of the mature osteoblasts treated with PTH.

## Materials and methods

### Animals

All transgenic mouse studies were approved by and performed in accordance with the Institutional Animal Care. Col1(2.3)-tTA/tetO-PTX^+^ mice (referred to as Col1(2.3)^+^/PTX^+^) and Col1(2.3)-tTA control littermates (referred to as WT) used in this study were generated by crossing mice that were true breeders for the Col1(2.3)-tTA transgene with mice heterozygous for the tetO-PTX transgene. Both of these mouse lines were generated in our laboratory and have been previously documented [11, 12].

The Col1(2.3)-tTA (line 139) mice carry a tetracycline transactivator (tTA) gene under the control of the osteoblast-specific Col1a1 2.3-kb promoter fragment, which drives the expression of the tTA protein specifically in osteoblasts. These Col1(2.3)-tTA mice have been described previously, and heterozygous Col1(2.3)-tTA mice have been shown to be phenotypically indistinguishable from wild-type mice [11].

The tetO-PTX mice express the catalytic subunit of pertussis toxin (PTX) under the control of the tTA-responsive tetO promoter [FVB-Tg(teto-ptxA)1Conk/Mmmh, MMRRC accession 014241-MU]. The Col1(2.3)^+^/PTX^+^ system operates as a Tet-OFF system, allowing reversible control of PTX expression in osteoblasts. PTX is a known inhibitor of Giα signaling.

To suppress PTX expression in Col1(2.3)^+^/PTX^+^ (referred to as PTX) mice, breeding pairs were maintained on a diet containing 200 mg/kg of doxycycline (DoxDiet; Bio-Serv, Frenchtown, NJ, USA) from conception until 16 weeks of age. The control Col1(2.3)-tTA (WT) littermates were also maintained on the same doxycycline-containing diet. To induce PTX expression in osteoblasts, the animals were then switched to a standard chow diet prior to the initiation of PTH administration.

To profile the transcriptome of the PTX^+^ osteoblasts with gene microarray, we co-expressed a histone-GFP marker in osteoblasts in vivo alone (abbreviated Col1(2.3)^+^/GFP^+^) or with PTX in triple transgenic mice (abbreviated Col1(2.3)^+^/GFP^+^/PTX^+^). This was accomplished by generating mice harboring a TetO-histone-GFP gene (mouse line Tg (TetO-HIST1H2BJ/GFP) 47Efu/J, Jackson Laboratory) with a tetO-PTX gene. Double transgenic mice were then crossed with mice homozygous for the Col1(2.3)-tTA genes to examine the skeletal effects of Gi signaling in osteoblasts. All animals in this study were generated using FVB/N oocytes and thus are maintained on the same background. Mice, except for those used for osteoblast isolation, were fed a diet containing 200 mg/kg of doxycycline to suppress transgene activity since conception [11, 17]. Previously, we reported a sex dimorphic expression of the PTX transgene, and thus, we did not observe a bone phenotype in the male transgenic mice during aging [13]; therefore, we only included adult female mice in this study.

### Intermittent Injection of Recombinant Parathyroid Hormone (iPTH)

**Fig. 1:**
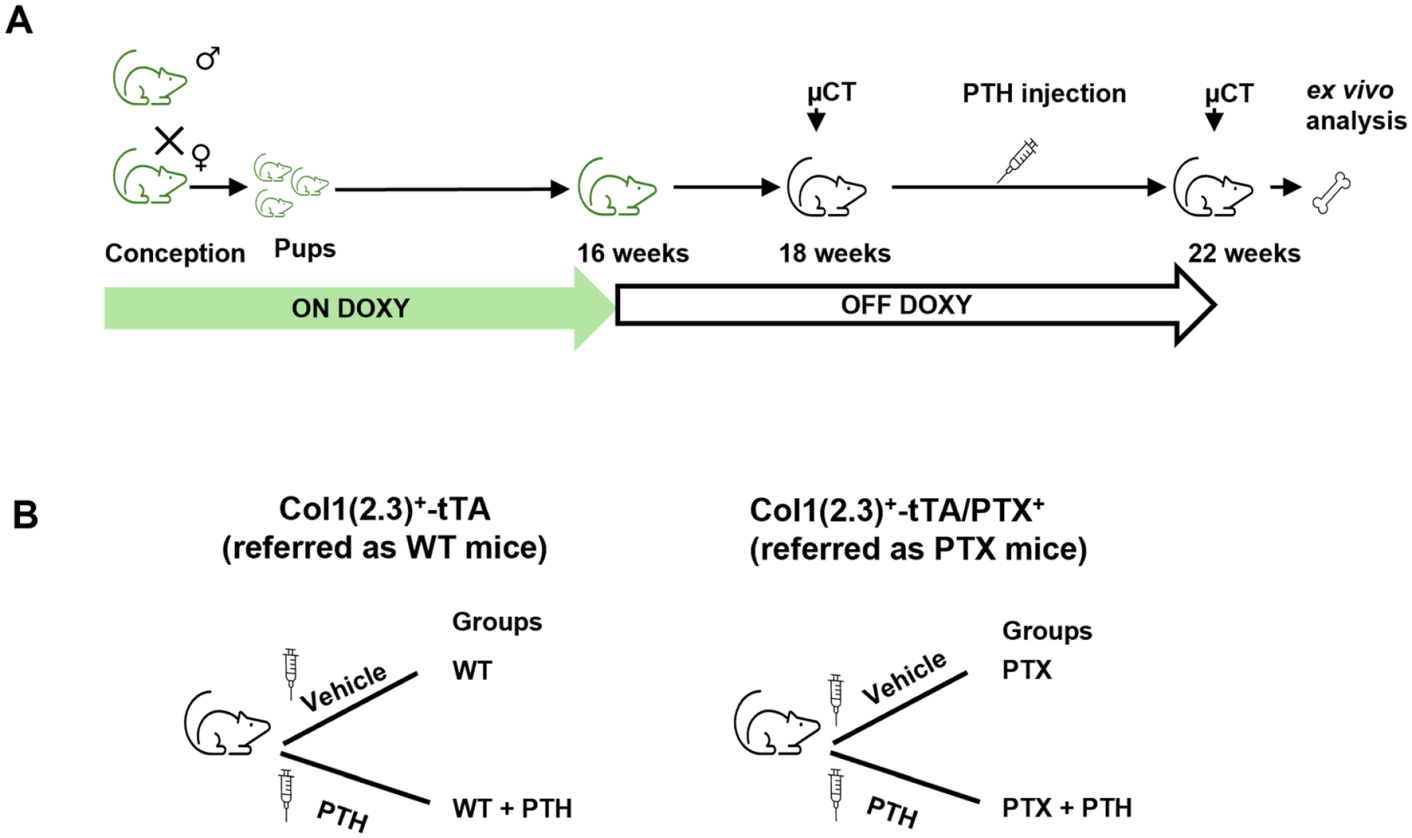
Experimental design to study the effect of inhibition of Gi signaling in vivo on the anabolic action of PTH. **A** Schematic showing treatment schedule and **B** the experimental design in control Col1(2.3)^+^-tTA mice (referred as WT), and Col1(2.3)^+^-tTA/PTX^+^ (referred as PTX) littermates. ON-DOXY, maintained on doxycycline-containing diet; OFF-DOXY, maintained on normal diet

Female Col1(2.3)^+^/PTX^+^ and the littermate Col1(2.3)^+^tTA control mice (referred to as WT here after) at 4.5 months of age were injected with PTH (Fig. 1A). Two weeks prior to treatment, the mice were maintained off doxycycline-containing diet and switched to a normal mouse chow (OFF-DOXY) and treated with either PTH or solvent vehicle. The mice were randomly grouped as (1) WT + vehicle, (2) WT + PTH, (3) PTX + vehicle, and (4) PTX + PTH (Fig. 1B). The number of animals in each group was given by combining litters generated from the same breeding pair. Recombinant human PTH [1–34] (Bachem Inc., CA) was dissolved in acid PBS (10 mM acetic acid and 1xPBS) with 2% heat inactivated C57BL/6 serum. The PTH and vehicle solutions were aliquoted and stored in − 20 °C freezer. The freshly thawed PTH was injected subcutaneously at a dose of 80 μg/kg body weight, between 10 and 12 AM, 5 consecutive days per week, for 4 weeks. WT mice were given the same volume of vehicle–acid PBS (10 mM acetic acid and 1xPBS) with 2% heat inactivated C57BL/6 serum. Demeclocycline (15 mg/kg body weight; Sigma) and calcein (20 mg/kg body weight; Sigma) were injected i.p. 2 and 7 days, respectively, before euthanasia.

### Micro-Computed Tomography (µCT) in vivo

µCT in vivo scans were performed prior to PTH administration and at the end of the experiment. The distal femur and tibio-fibular junction (TFJ) were scanned by a vivaCT-40 µCT system (Scanco Medical AG, Bruttisellen, Switzerland). All in vivo μCT images were obtained using an X-ray energy of 55 kV with a voxel size of 10.5 μm and an integration time of 1000 ms. The cancellous bone in the distal femur was assessed from a total of 100 scan slices, starting 0.3 mm away from the growth plate (threshold = 0.8/1/270) [13]. Quantitative assessment of the diaphyseal cortex at TFJ was conducted using data from 40 slices (threshold = 0.8/1/365) [13].

### Histomorphometry

After euthanizing mice, the left femur, tibia, and the fourth lumbar vertebral bone (L4) were collected, cleaned of surrounding tissues, and fixed in 10% phosphate-buffered formaldehyde solution (Fisher Scientific, Pittsburgh, PA, USA) for 48 h at 4 °C. Bones were then transferred into 70% ethanol and stored in the dark at 4 °C until histomorphometry. The femur and the L4 vertebrae stored in ethanol were embedded in methyl methacrylate without decalcification and sectioned with Jung 2065 and 2165 microtomes (Leica, 145 Bannockburn, IL, USA). The 5-μm-thick longitudinal sections from the distal femur and the L4 were subjected to Von Kossa/Trichrome staining. Assessment of bone mineralization and formation was performed on the 10-μm-thick, unstained sections. Mosaic-tiled images of cancellous bone and cortical bone were acquired at X 200 with a Zeiss Axioplan Imager M1 microscope fitted with a motorized stage (Carl Zeiss MicroImaging, Thornwood, NY, USA). The tiled images were then stitched and converted to a single image using the Axiovision software (Carl Zeiss MicroImaging) prior to histomorphometry using a Bioquant Image Analysis System (Nashville, TN, USA). Cancellous bone was assessed in a region of 100 µm from the lowest point on the growth plate, extending 1 mm down the metaphysis. The dynamic indices of bone formation that were measured include mineralizing surface (MS/BS), mineral apposition rate (MAR), and surface-based bone formation rate (BFR/BS) [13].

### Serum Chemistry

Blood was collected from mice at the time of euthanasia. Serum was extracted using microtainer serum separator tubes (BD Biosciences, San Jose, CA, USA). Serum procollagen type I amino-terminal propeptide (PINP) and serum pyridinoline (PYD) were quantified using the rat/mouse PINP EIA Kit AC-33F1 from Immunodiagnostic Systems (Scottsdale, AZ, USA) and the Metra Biosystems Serum PYD Kit 8019 (Metra Biosystems, Santa Clara, CA, USA) according to the manufacturers’ directions [18].

### Biomechanical Testing

Load-to-failure test was performed to determine whole bone behavior at anatomic sites consisting of cortical bone (femoral diaphysis). In brief, the right femurs were subjected to a 3-point bending test [19]. Each femur was placed on two supports (7 mm span) and a transverse load was applied to the mid-diaphysis under displacement control with a rate of 0.03 mm/s (20). All tests were performed at room temperature using a voice coil-based load frame (ElectroForce 3200; Bose, Eden Prairie, MN). Femoral strength was defined as the maximum force sustained during the tests.

### Isolation of Mouse Calvarial Osteoblasts

To isolate osteoblasts, mice were maintained on regular chow without doxycycline administration to allow transgene expression in experimental mice since conception. Osteoblasts were isolated from 1-month-old mice calvariae [21]. In brief, calvariae from Col1(2.3)^+^/GFP^+^/PTX^+^ or Col1(2.3)^+^/GFP^+^ mice were isolated, cleaned of surrounding tissues, and subjected to four sequential digestions in an enzymatic mixture (1.5 U/ml collagenase P, 0.05% trypsin and 0.25 mM EDTA in PBS). Cell fractions 2–4 were collected, pooled, and sorted [21].

Fluorescent-activated cell sorting (FACS) was performed using a BD FACS Aria sorter (BD Biosciences) at the SFVAMC Cell Sorting Core facility. Cells were gated for GFP signals and sorted through a 100-mm nozzle. A fraction of the sorted cell population (5 × 10^3^) was used for FACS reanalysis by the same gating strategy to assure the maximum purity. Cell suspensions were kept cold during the entire sorting process to minimize changes in gene expression [21].

### Microarray Analysis

The FACS-sorted GFP^+^ cell populations were subjected to RNA extraction immediately after sorting. RNA was purified using the Arcturus PicoPure™ RNA isolation kit (Applied Biosystems, Carlsbad, CA) and then treated with DNase with the Qiagen DNase Set (Valencia, CA). The quantity and quality of total RNA were determined by Agilent 2100 Bioanalyzer (Agilent Technologies). The 28S/18S ratios of the RNA samples were in the range of 1.8–2.1, and their RNA integrity numbers were in the range of 8.8–10. Reverse transcription and amplification of isolated RNA into cDNA were performed using the NuGEN FFPE WTA kit (NuGEN, San Carlos, CA). The integrity of resultant cDNA was assessed using the Agilent 2100 Bioanalyzer and individual samples were further processed and hybridized to Affymetrix Mouse Gene 1.0 ST arrays (Affymetrix, Cleveland, OH) before scanning, according to the protocol in WT Sense Target Labeling Assay Manual from Affymetrix (Version 4; FS450_0007) at the UCSF Gladstone Genomics Core Facility. Expression analysis was performed on three separate osteoblast preparations of RNA from each genotype. Data were normalized using Guanine Cytosine Robust Multi-Array Analysis (GC-RMA). Comparison groups were annotated with statistically significant Gene Ontology (GO) term overrepresentation using the GO-Elite software packages. Gene set enrichment analysis (GSEA) is performed using the GSEA analysis software v4.1.0.

### Real-time PCR (qPCR)

Epiphyses were removed from the freshly isolated long bones (both humerus and right tibia) and kept frozen in liquid nitrogen after bone marrow was flushed out. Upon RNA extraction, the frozen bones were pulverized in RNA STAT60 using a biopulverizer (Biospec Products, Inc., Bartlesville, OK, USA). RNA was extracted with chloroform and alcohol and then purified using PureLink™ RNA Mini Kit. cDNA was synthesized using Applied Biosystems TaqMan Reverse Transcription Reagents (Foster City, CA, USA). Gene amplification was performed using SYBR Green master mixes and measured using ABI ViiA™ 7 real-time system (Thermo Fisher Scientific, Inc.). Analysis was carried out using the SDS software supplied with the system. Target gene expression was normalized to the house keeping gene, *Gapdh*, within the same sample [13, 18]. Target genes and the primers used are listed in Table 1.

**Table 1:** Primer list for qPCR

Gene	Description	Forward primer	Reverse Primer
*Col1*	Collagen Type I	GCG AAG GCA ACA GTC GCT	CTT GGT GGT TTT GTA TTC GAT GAC
*Ocn*	Osteocalcin	CTG ACC TCA CAG ATG CCA AG	GTA GCG CCG GAG TCT GTT C
*Runx2*	RUNX2	CGA GAC CAA CCG AGT CAT TT	ACG CCA TAG TCC CTC CTT TT
*Osx*	Osterix	TTT CTC ATT AAC TCG TTG CCA TCT	CTT CGG GAA AAC GGC AAA TA
*Tnfsf11*	RANKL	TTG CAC ACC TCA CCA TCA AT	TCC GTT GCT TAA CGT CAT GT
*Tnfrsf11b*	OPG	CAG AGA CCA GGA AAT GGT GAA	AAG CTG CTC TGT GGT GAG GT
*Dkk1*	Dickkopf 1	CCG GGA ACT ACT GCA AAA AT	CGT TGT GGT CAT TAC CAA GG
*Gapdh*	GAPDH	TGC ACC ACC AAC TGC TTA G	GGA TGC AGG GAT GAT GTT C

### Statistical Analysis

All data collected from the PTH or vehicle-treated WT and PTX mice are presented as means ± SEM. Statistical significance was ascertained between all the groups using two-way ANOVA with post hoc Tukey’s test. Statistical significance was taken as *p* < 0.05. For microarray analysis, Bayesian statistical analysis was carried out using Linear Models of Microarrays (LIMMA) to identify statistically significant differentially expressed genes between Col1(2.3)^+^/GFP^+^ and Col1(2.3)^+^/GFP^+^/PTX^+^ osteoblasts. Moderated *t-*statistics and the associated *p*-values were calculated and *p-*values less than 0.01 were considered statistically significant.

## Results

### PTH Increases Bone Mass in PTX Mice

**Fig. 2:**
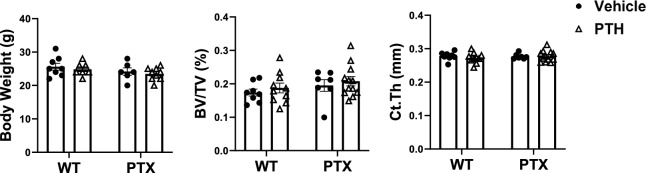
Baseline measurements of body and bone mass prior to PTH administration in mice. **A** basal body weight, *n* = (6–9/group) **B** cancellous fractional volume (BV/TV) at distal femur, and **C** cortical thickness (Ct.Th) at tibio-fibular junction (TFJ) in the littermate wild-type and Col1(2.3)^+^/PTX^+^ (PTX) mice before intermittent PTH administration. For Figure B and C, (WT + Vehicle, *n* = 8; WT + PTH, *n* = 10; PTX + Vehicle, *n* = 7; PTX + PTH, *n* = 12)

To evaluate if an active Gi signaling in adult mice osteoblasts limits the anabolic action of PTH, we used a Tet-OFF transgenic mouse line-Col1(2.3)-tTA/tetO-PTX, which ensures PTX expression in the absence of doxycycline, and specifically in mature osteoblasts as it is under the control of Col1(2.3) promoter. *Col1(2.3)*^+^*/PTX*^+^ (labeled as PTX mice) and their littermates *Col1(2.3)*^+^*-tTA* (labeled as WT) mice were maintained on Doxy diet for 4 months since conception to inactive the expression of PTX in the pups. Two weeks before PTH injection, the expression of PTX transgene was activated by switching to regular chow (Fig. 1A). Body weights of these mice before PTH started at a same level (Fig. 2A). µCT in vivo scan showed all groups had an equal basal cancellous bone fractional volume (BV/TV) at the distal femur and an equal cortical thickness (Ct.Th) at TFJ prior to PTH injection (Fig. 2B and C).

**Fig. 3:**
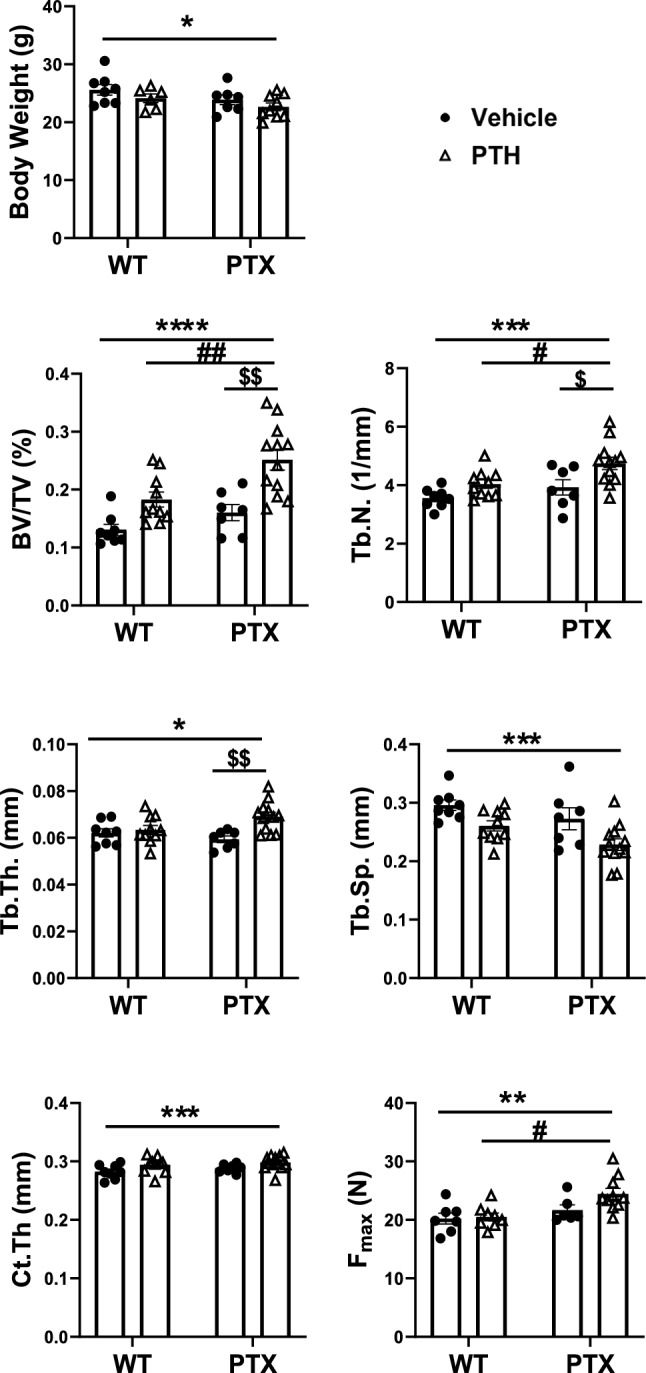
PTX mice display higher bone mass and strength upon PTH administration. **A** Body weights, in vivo μCT assessing cancellous bone at distal femur (**B**), and cortical bone at TFJ (**C**). **D** mechanical strength (Fmax) at the midshaft of femur was determined using three-point bending. *TV* tissue volume, *BV* bone volume, *BV/TV* bone fractional volume, *Tb.N* trabecular number, *Tb.Th* trabecular thickness, *Tb.Sp* trabecular separation, *Ct.Th* cortical bone thickness. *p < 0.0332, **p < 0.0021, ***p < 0.0002, ****p < 0.0001 vs. vehicle-treated WT littermates; #*p* < 0.0332, ##*p* < 0.0021 vs. WT + PTH; $*p* < 0.0332, $$p < 0.0021 vs. PTX + Vehicle. Data are represented as mean ± SEM and statistical significance was calculated using two-way ANOVA. (WT + Vehicle, *n* = 8; WT + PTH, *n* = 10; PTX + Vehicle, *n* = 7; PTX + PTH, *n* = 12)

At the end of the experiment, most of the mice had almost the same body weights as the controls (WT) except for the PTX + PTH, which showed a slight decrease as compared to vehicle treated WT(Fig. 3A). A live µCT scan was performed at the distal femur and TFJ. µCT assessment demonstrated that iPTH injection significantly increased BV/TV by 49.4% and Tb.N by 13.6% and decreased Tb.Sp by 11.9% in the distal femur in WT mice (Fig. 3B). The expression of the PTX gene alone significantly increased BV/TV by 31% and Tb.N by 10.5% in PTX mice, compared to the vehicle-treated wild-type mice (WT). iPTH and blocking G_i_ produced an additive effect on the bone fractional volume of PTX + PTH, in which iPTH increased BV/TV by 111.8%, Tb.Th by 10.6%, and Tb.N by 36.5%, and decreased Tb.Sp by 25% (Fig. 3B). At TFJ, the expression of PTX gene alone or iPTH injection had no effects on Ct.Th. In contrast, PTH injection significantly increased Ct.Th by 5.6% in the Col1(2.3)^+^/PTX^+^ mice (Fig. 3C). Furthermore, the three-point bending test to assess the mechanical strength of the femur showed no difference in WT vs. WT + PTH or PTX. However, iPTH significantly increased the mechanical strength at the midshaft of the femur in PTX mice (Fig. 3D).

**Fig. 4:**
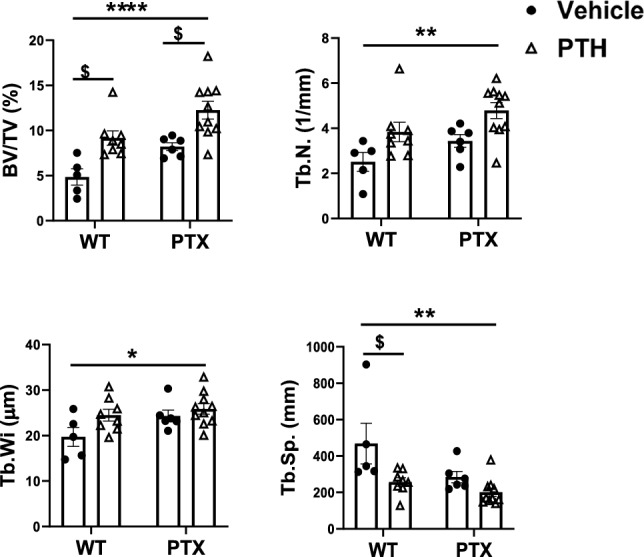
Static histomorphometry analysis confirms higher bone mass in PTX mice upon PTH administration Static histomorphometry of cancellous bone at left distal femur in and littermate control mice. *TV* tissue volume, *BV* bone volume, Bone fractional volume *BV/TV*, *Tb.N* trabecular number, *Tb.Wi* Trabecular width, *Tb.Sp* trabecular separation. Data are represented as mean ± SEM and statistical significance was calculated using two-way ANOVA. *p < 0.0332, **p < 0.0021, ****p < 0.0001 vs. vehicle-treated WT littermates; $ *p* < 0.0332 vs. PTX + Vehicle. (WT + Vehicle, n = 5; WT + PTH, *n* = 8; PTX + Vehicle, *n* = 6; PTX + PTH, *n* = 10)

Static histomorphometry of the cancellous bone at the distal femur confirmed the findings by μCT (Fig. 4). The effect of PTH on the lumbar vertebra (L4) was also assessed by histomorphometry (Suppl. Figure 1) [22]. Activation of PTX transgene expression alone had no effect on the vertebral BV/TV in PTX mice. But iPTH significantly increased BV/TV and showed a trend of improved Tb.N and decreased Tb.Sp in both wild-type and PTX mice (Suppl. Figure 1) [22]. Moreover, the increment of BV/TV in PTX + PTH was significantly higher than that of the WT + PTH or vehicle-treated PTX group (Suppl. Figure 1) [22].

### PTH Increases Bone Formation in PTX Mice

**Fig. 5:**
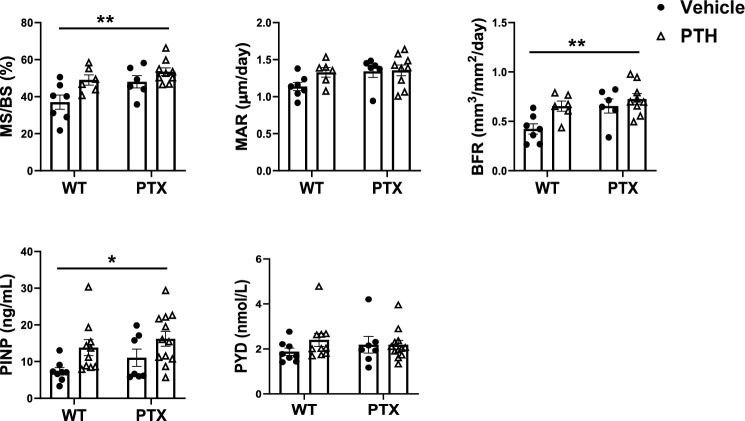
Dynamic histomorphometry shows higher bone formation in PTH treated PTX mice. **A** Mineralizing surface (MS/BS), mineralization appositional rate (MAR), and bone formation rate (BFR) were determination by dynamic histomorphometry at the distal femur. (WT + Vehicle, n = 7; WT + PTH, *n* = 6; PTX + Vehicle, *n* = 6; PTX + PTH, *n* = 9). **B** Bone formation and resorption were determined by measuring serum markers PINP and PYD. Data are represented as mean ± SEM and statistical significance was calculated using two-way ANOVA. (WT + Vehicle, *n* = 8; WT + PTH, *n* = 10; PTX + Vehicle, *n* = 7; PTX + PTH, *n* = 12). *p < 0.0332, **p < 0.0021 vs. vehicle-treated WT littermates

To investigate the mechanism of how endogenous G_i_ signaling limits bone response to iPTH, bone mineralization and bone formation was determined at the distal femur. Compared to vehicle-treated WT, iPTH treatment showed a trend of increased cancellous BFR and MAR in wild-type mice (WT + PTH) and in Col1(2.3)^+^/PTX^+^ mice (PTX + PTH) (Fig. 5A). It was also noted that, in addition to significantly increase MS/BS, MAR, and BFR in the vehicle-treated PTX, OFF-DOXY enhanced the stimulatory effect of iPTH on cancellous MAR and BFR in PTX + PTH (Fig. 5A).

Compared to WT, iPTH treated mice displayed increased PINP, a marker of bone formation, in both wild-type and Col1(2.3)^+^/PTX^+^ mice (Fig. 5B**)**. However, the expression of the PTX gene alone had no effect on the level of PINP in Col1(2.3)^+^/PTX^+^ mice. PYD, a marker of bone resorption, did not show any significant difference between any groups in this study (Fig. 5B).

Real-time PCR demonstrated that iPTH increased the mRNA levels of osteoblastic marker genes, encoding collagen type I, osteocalcin, and Runx2, in both wild-type and Col1(2.3)^+^/PTX^+^ mouse bones (Suppl. Figure 2) [22]. Additionally, iPTH upregulated the gene expression of Tnfsf11, which encodes for RANKL, and increased the ratio of Tnfsf11/Tnfrsf11b in Col1(2.3)^+^/PTX^+^ mice (Suppl. Figure 2) [22]. In this study, iPTH increased the level of *Dkk1* mRNA, but had no effect on the expression of *Sost* gene (data not shown), in both wild-type and Col1(2.3)^+^/PTX^+^ littermates (Suppl. Figure 2) [22].

### Identifying Genes Differentially Expressed in Col1(2.3)^+^/PTX^+^ Mouse Osteoblasts

**Fig. 6:**
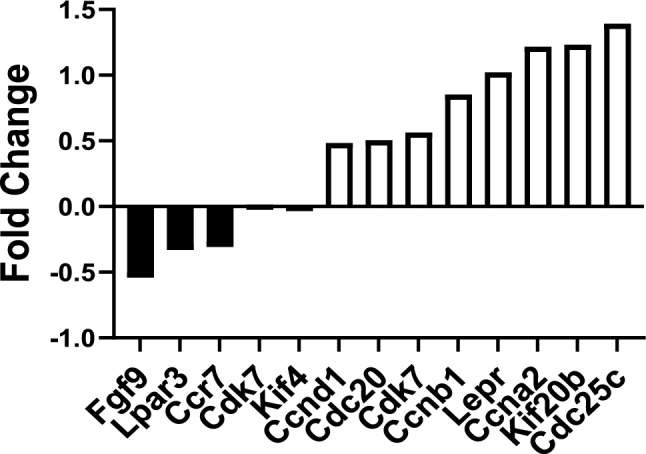
Microarray analysis displays differentially regulated genes in osteoblasts derived from PTX mice. Graphic representation of selected gene expression altered by blocking endogenous Gi signaling in Col1(2.3)^+^/GFP^+^/PTX^+^ mouse osteoblasts. Data are represented as fold change vs. control mice

We performed microarray gene expression profiling on the purified Col1(2.3)^+^/GFP^+^/PTX^+^ mouse calvarial osteoblasts. Based on a cut-off of false discovery rate (FDR) of 0.1, we identified 3527 transcripts out of about 28,598 genes present on the array that were significantly modulated by G_i_ signaling. We applied the Gene Set Enrichment Analysis (GSEA) to identify any overrepresented predefined gene sets in PTX^+^ cells. At *p* < 0.05, GSEA showed 31 gene sets are upregulated by PTX expression, among which 12 gene sets are significantly enriched at *p* < 0.05 (Table 2). These results indicated that blocking G_i_ signaling stimulated the proliferation of osteoblasts by regulating genes that are involved in the cell division cycle (Table 2). *Ccna2*, *Ccnb1*, *Ccnd1*, *Cdc20*, *Cdc25c*, *Cdk7*, *Kif20b*, and *Kif4* were a few of the top most upregulated genes. We also identified that the expression of some stem cell maker genes and growth factors was altered by G_i_ blocking. The Leptin receptor (*Lepr*) was upregulated and fibroblastic growth factor 9 (*Fgf9*) was downregulated. G_i_-coupled receptors (*Ccr7*, *Lpar3*) were downregulated by G_i_ blocking in osteoblasts as well (Fig. 6).

**Table 2:** Summary of GSEA results with enrichment scores

Gene Set	Enrichment Score	Normalized Enrichment Score	Normalized p-Value
HALLMARK_G2M_CHECKPOINT	0.66	3.67	0.000
HALLMARK_E2F_TARGETS	0.65	3.62	0.000
HALLMARK_MITOTIC_SPINDLE	0.63	3.27	0.000
HALLMARK_MYC_TARGETS_V1	0.64	3.23	0.000
HALLMARK_MTORC1_SIGNALING	0.60	3.00	0.000
HALLMARK_OXIDATIVE_PHOSPHORYLATION	0.64	2.86	0.000
HALLMARK_DNA_REPAIR	0.58	2.28	0.000
HALLMARK_UNFOLDED_PROTEIN_RESPONSE	0.50	1.97	0.007
HALLMARK_GLYCOLYSIS	0.42	1.92	0.000
HALLMARK_INTERFERON_GAMMA_RESPONSE	0.43	1.71	0.032
HALLMARK_ADIPOGENESIS	0.35	1.64	0.029
HALLMARK_PEROXISOME	0.44	1.62	0.037
HALLMARK_PI3K_AKT_MTOR_SIGNALING	0.37	1.57	0.052
HALLMARK_ESTROGEN_RESPONSE_LATE	0.35	1.47	0.084
HALLMARK_FATTY_ACID_METABOLISM	0.34	1.47	0.078

## Discussion

The anabolic effect of intermittent PTH (iPTH) therapy on bone has been demonstrated in both humans and mice. However, the anabolic action of PTH is blunted by some factors [23–26]. Given PTH activates G_s_ and G_i_ pathway by acting through the PTH/PTHrP receptor 1 (PTHR1), the downstream effectors of G_i_ activation would antagonize G_s_-mediated increase in adenylyl cyclase activity in osteoblasts and limit bone response to PTH. Our previous study using Col1(2.3)tTA–tetO-PTX mice has shown that endogenous G_i_ signaling restrains bone formation and produces an obvious mouse bone phenotype at the age of 3 months [12]. We chose 4-month-old mice in this study because we expected an accumulated “G_i_ tone” within osteoblasts which would significantly blunt the bone anabolic effect of PTH in mice at 4 months of age or older. We have used the same Col1(2.3)tTA–tetO-PTX mice to evaluate whether inhibiting endogenous Gi signaling can help augment the anabolic action of PTH. Col1(2.3)tTA–tetO-PTX mice is Tet-OFF mouse system, which offers spatio-temporal regulation of PTX expression, which is a well-known Giα signaling inhibitor. In the presence of doxycycline (a tetracycline), given in the diet in our study, the expression of PTX is blunted, whereas the PTX expression resumes when the mice are switched to normal chow diet. Two weeks after switching the diet, recombinant PTH at a dose of 80 µg/kg body weight was administered to both PTX and WT littermates for 5 days/week for a duration of 4 weeks. This dosage regimen of PTH is known to cause bone anabolic action [26]. We found that PTH administration and Gi inhibition in PTX mice displayed moderate anabolic effects at both the femur and lumbar vertebral bones in WT mice, using µCT. Intriguingly, PTH treatment in PTX mice significantly enhanced cancellous bones and moderately enhanced the cortical bones as compared to WT mice treated with PTH and PTX transgenic mice. These results were corroborated by static histomorphometric analysis at the femur, where PTH administration showed significant increase in bone mass in WT mice, as expected, and that the bone mass was significantly higher in the PTX upon PTH administration as compared to WT treated with PTH. These results support our hypothesis that activated G_i_ signaling in osteoblasts limits the anabolic effect of PTH in adult mouse bones. One of the strengths of our study is that this is a longitudinal study on mice to evaluate the role of Gi signaling in limiting the anabolic action of PTH.

Genetically manipulating G protein helps determine the roles of subclasses of G protein in bone homeostasis through characterizing their associated bone phenotypes. Specific activation of G_s_-coupled signaling in osteoblasts leads to a dramatic increase in trabecular bone formation with cortical bone resorption in transgenic mice (RS1 mice) [27]. In contrast, blockade of the receptor-activated G_i_ signaling in osteoblasts leads to an increase in both cortical bone and cancellous bone formation, which does not completely replicate the bone phenotype of RS1 mice [27, 28]. One of the possible mechanisms is that activation of Gs or blocking Gi signaling in osteoblasts regulates different autocrine or paracrine factors that affect bone homeostasis.

With the aid of microarray, we identified the cell proliferation-associated genes that encode *Ccna2*, *Ccnb1*, *Ccnd1*, *Cdc20*, *Cdc25c*, *Cdk7*, *Kif20b*, and *Kif4,* are highly expressed in the Col1(2.3)^+^/GFP^+^/PTX^+^ osteoblasts. Additionally, we found downregulation of *Fgf9* upon blockade of G_i_ signaling, that is known to negatively regulate bone mass by inhibiting osteogenesis [29, 30]. Moreover, it has been reported that activation of Gs signaling in osteoblast stimulates FGF9 expression. Furthermore, exposure of BMSC cultures with PTH or with forskolin markedly downregulated the expression of Fgf9 [21]. Taken together, these data indicated that one of the possible mechanisms by which endogenous G_i_ signaling limits the anabolic effect of PTH is possibly through regulating *Fgf9* expression in osteoblasts in adult mice.

There are few limitations of our study. The first being that the anabolic effect of PTH on WT mice and inhibition of Gi signaling itself on bone mass was not significant, at least in the µCT data, which partly due to small sample size and variations within the group. Nonetheless, the trend does depict that PTH and inhibition of Gi signaling in adult osteoblast increases bone mass and is clearly evident by static histomorphometry. Also, we performed this study in female mice as the inhibition of Gi signaling increases bone mass only in female mice (12, 13). Consequently, it underlines the need for studies in identifying factors limiting anabolic action of PTH in male mice. Another major limitation of our current study is that we have not performed any experiment to validate our microarray data on the osteoblasts from the PTX mice. Moreover, we have not made attempts to comprehensively decipher the mechanism(s) of Gi signaling limiting the bone anabolic actions of PTH. However, we did perform NGS RNA-Seq to get an unbiased view of transcriptome in Ocy454 cells treated with PTH (data not shown). Corroborating others’ finding, we observed that PTH upregulated some genes encoding factors that may have negative effects on bone. One such factor is sphingosine-1-phosphate (S1P) signaling (data not shown), which controls osteoclasts and bone homeostasis [31, 32]. S1P, acting through its G_i_ protein-coupled receptor (S1P1), is inversely correlated with bone mineral density, and positively correlated with bone resorption markers [14, 15]. Although blockade of G_i_ signaling in osteoblasts/osteocytes presumably prevents the bone loss mediated by G_i_ protein activation during PTH treatment, to what extent S1P inhibits bone formation is unknown. Similarly, our RNA-Seq data corroborates Wein et al. findings that PTH acts on osteocytes to increase HDAC4 (data not shown), which is reported to inhibit sclerostin expression [33].

In conclusion, endogenous Gi signaling in osteoblasts restricted bone anabolic function of iPTH in adult female mice and that blockade of this Gi signaling in osteoblasts with PTX increased the anabolic effects of PTH. Thus, the current study, and our on-going studies in the laboratory, where we are investigating the crosstalk between PTH and Gi signaling pathways, will open new avenues for therapeutic targets in treating osteoporosis.

## Supplementary Information

Below is the link to the electronic supplementary material.

Supplementary file1 (TIF 240 KB)

Supplementary file2 (TIF 335 KB)

## Data Availability

Some or all datasets generated during and/or analyzed during the current study are not publicly available but are available from the corresponding author on reasonable request.

## Declarations

### Research involving Human and animal participants

All transgenic mouse studies were approved by and performed in accordance with the Institutional Animal Care and Use Committee at the Veterans Affairs Medical Center, San Francisco (SFVAMC).
