# Supplementary figures and images for: Blockade of Gi Signaling Enhances the Anabolic Effect of Parathyroid Hormone in Female Mice
Source: Calcif Tissue Int. 2025 Jul 16;116(1):98. doi: 10.1007/s00223-025-01409-2 (PMC12267360; doi:10.1007/s00223-025-01409-2)

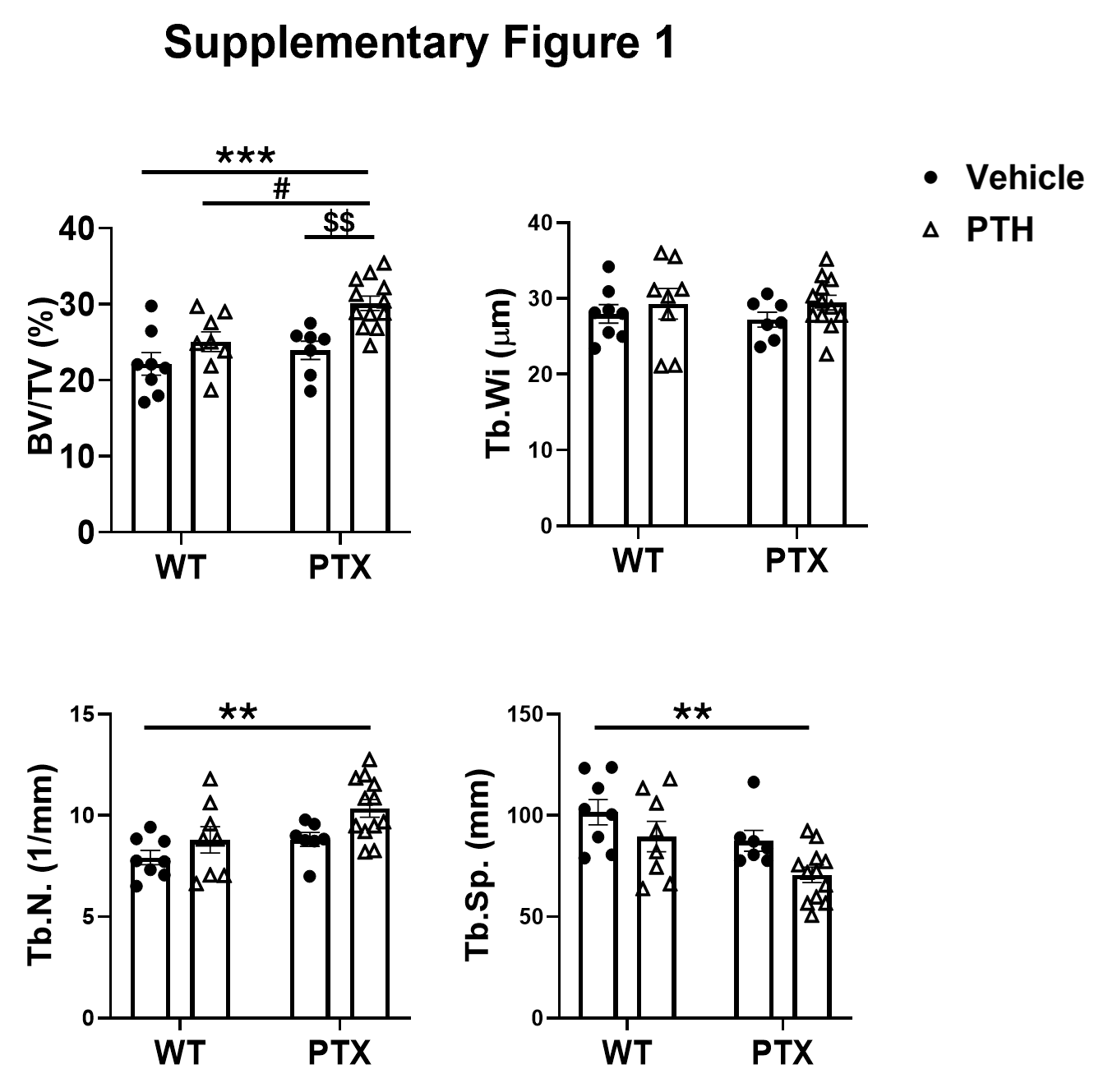

Supplement: Supplementary file 1 — Supplementary file1 (TIF 240 KB) [file 223_2025_1409_MOESM1_ESM.tif]

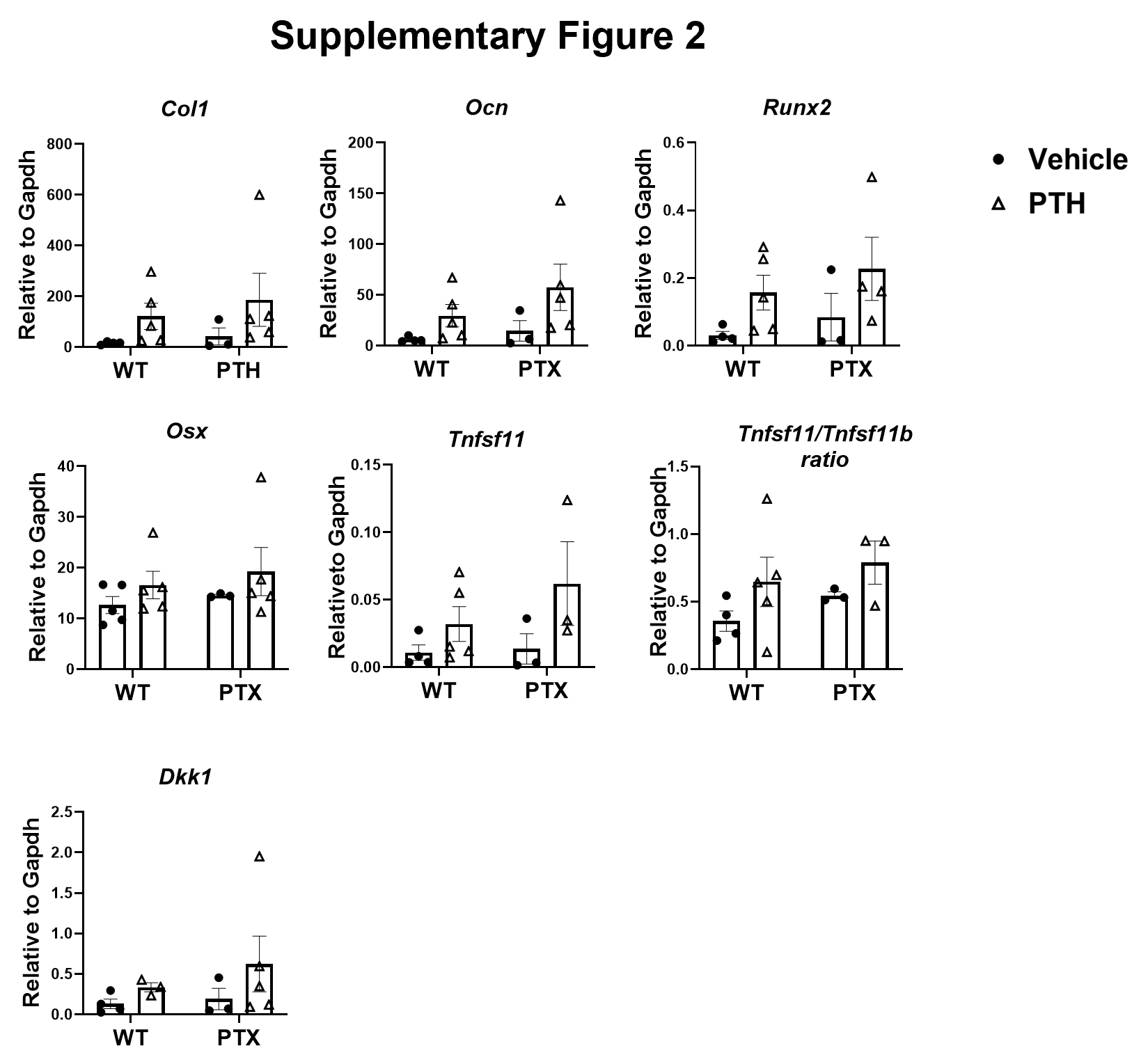

Supplement: Supplementary file 2 — Supplementary file2 (TIF 335 KB) [file 223_2025_1409_MOESM2_ESM.tif]
